# Development and application of a couple-centered antenatal education program in Korea

**DOI:** 10.4069/kjwhn.2021.06.20

**Published:** 2021-06-29

**Authors:** Minseon Koh, Jisoon Kim, Hyeji Yoo, Sun A Kim, Sukhee Ahn

**Affiliations:** 1 College of Nursing, Yeoju Institute of Technology, Yeoju, Korea; 2 Department of Nursing, Woosong University, Daejeon, Korea; 3 College of Nursing, Chungnam National University, Daejeon, Korea

**Keywords:** Marriage, Parents, Pregnant woman, Prenatal education, Spouses

## Abstract

**Purpose:**

This study was conducted to develop a couple-centered antenatal education program and to test the program’s feasibility.

**Methods:**

With a preliminary-experimental study design, 33 pregnant couples who were expecting their first child participated in this study. The program consisted of four sessions (1 hour/session/week) of education and counseling. Data were collected before and after the intervention from September 2018 to April 2019 at a women’s hospital in Daejeon, Korea, with demographic data forms, the Edinburgh Postnatal Depression Scale, Perceived Stress Scale, Maternal–Fetal Attachment Scale, Korean Newborn Care Confidence Scale, Wijma Delivery Expectancy/Experience Questionnaire, and Dyadic Adjustment Scale-10.

**Results:**

The pregnant women and their husbands were on average 32.30±3.10 and 33.21±6.25 years old, respectively. The mean marriage duration was 2.34±1.63 years, the gestational age was 31.30±2.66 weeks, and 78.8% of the couples had a planned pregnancy. After the program, both the pregnant women and their husbands showed significant improvements in attachment to the fetus and confidence in providing infant care. Prenatal depression, prenatal stress, and fear of childbirth in pregnant women significantly decreased after completing the program. However, the dyadic adjustment score did not change significantly either in the pregnant women or their husbands.

**Conclusion:**

A couple-centered antenatal education program seems to be effective for couples adjusting to parenthood, but further studies should explore ways to have a positive impact on couples’ relationships.

## Introduction

Parenthood begins with having the first child; pregnancy is a time of preparation, and the postpartum period is a time of change in the marital relationship due to the new parental role [1]. Anxiety, depression, and changes in marital relationships during pregnancy are associated with the fear of childbirth [2] and an increased risk of preterm birth and low birthweight [3]. The needs and challenges, birth experiences, and changes in roles and relationships of pregnant women cause maternal stress [4]. In their husbands, negative emotions and fears related to the father identity and father role lead to stress [5], and mothers and fathers adapt differently to parenthood due to their different experiences of pregnancy and childbirth [6]. Therefore, prenatal education programs need to provide information for both prospective fathers and mothers [7].

Worsening of the marital relationship has a negative effect on attachment to the fetus, as well as the process of adapting to changes in the marital relationship during the transition period [8]. Conversely, higher satisfaction in the marital relationship during pregnancy and maintenance of a good relationship improve adaptation to the parental role in the postpartum period [9]. Preparing for the parental role together and maintaining the marital relationship facilitate the parental transition process [10]. In a gender-equal society, there is little difference in the roles of mothers and fathers as parents; therefore, gender-equal prenatal education should be provided when fathers participate in pregnancy and childrearing [11,12].

Mercer [13] theorized that during the process of becoming a mother, the family is a dynamic system that includes mother–father, maternal-fetal, and paternal-fetal interactions, and that the role of the mother is affected by the interactions of the mother with the father and baby. However, this framework leaves the father as a supporter to help his partner become a mother, overlooking the process of becoming a father; this has led to a tendency for husbands to be alienated from prenatal education [13]. The process of parental change is an experience shared by couples [14] and an inter-couple process [15], meaning that a comprehensive approach is needed in the process of becoming parents for both pregnant women and their husbands. Therefore, the theory of becoming a mother by Mercer [13] needs to be expanded to an approach to becoming parents that includes both spouses. In addition, prenatal education that includes communication and mutual support is effective in promoting adaptation in the marital relationship during the transition period [16]. A recent meta-analysis of the effects of psychoeducation for couples combining the parental aspect and couple-relationship education also reported that such interventions reduce negative effects, such as postpartum depression, on the maternal and paternal side, and improve couple-relationship satisfaction [17]. The authors argued that couple-centered prenatal education is more effective if it combines elements of the parental role and marital relationship [16].

**Figure 1.**
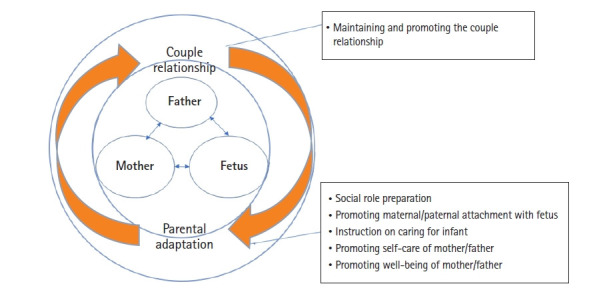
Conceptual framework of pregnant couple’s becoming parents.

Most prenatal education in South Korea (hereafter, Korea) is provided for only pregnant women (64.4%), with a focus on delivery, breastfeeding, and infant care; meanwhile, very few studies have explored interventions dealing with mother–father interactions or couple relationships [18], leaving a gap in the literature that needs to be addressed. Interventions for pregnant couples who are transitioning to parenthood are needed to help couples adapt to becoming parents and to address the couple relationship. The purpose of this study was to develop a couple-centered antenatal education program to help couples better adapt to becoming parents and to address aspects of the couple relationship, and then to confirm its effectiveness and applicability based on Mercer’s theory of becoming a mother [13] (Figure 1).

The hypotheses of this study were as follows.

1) Pregnant couples who participate in the couple-centered antenatal education program will have a better adaptation to becoming parents, as shown by measures including antenatal depression, antenatal stress, fetal attachment, newborn-care confidence, and mother’s fear of childbirth.

2) Pregnant couples who participate in the couple-centered antenatal education program will improve their adaptation to the couple relationship, as measured by the couple’s dyadic adjustment.

## Methods

Ethics statement: This study approved was by the Institutional Review Board of Chungnam National University (201806-SB-063-01). Informed consent was obtained from the participants.

### Study design and setting

**Figure 2.**
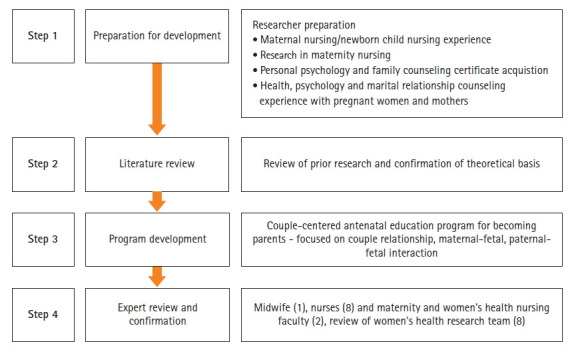
Development process of the couple-centered antenatal education program.

The couple-centered antenatal program was developed by the first author through multiple stages of program development, as shown in Figure 2. This program was delivered by the first author and a midwife at a women’s hospital in Daejeon, Korea, with a single-group pretest-posttest study design from September 2018 to April 2019. A member of the research team explained the study and the possibility of participation to couples in the outpatient waiting room. If they voluntarily agreed to participate, they received information about the program schedule, a preliminary survey was conducted, and a follow-up investigation was conducted immediately after they finished the fourth session of the program (1 hour/session/week).

### Participants

The study participants were married couples, including women who were pregnant with their first child at 28-34 weeks of gestation, because couples who answered that they were well prepared in the third trimester were found to adapt well in the postpartum period [19]. Since this antenatal educational program targeted low-risk segments of the general population, pregnant women who had complications (such as gestational diabetes, hypertension, or placental and cervical problems) or multiple pregnancies were excluded; additionally, couples were excluded if either the pregnant woman or the husband had any other health problems.

**Figure 3.**
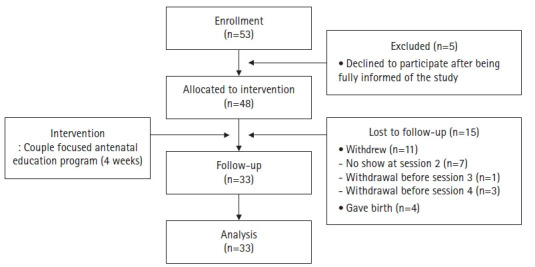
Flow diagram of the study.

The required sample size was calculated based on effect sizes (d) of 1.71 and 0.47 for a childbirth education pilot study aimed at preventing fear of childbirth and depression, respectively, among pregnant women [20]. For the effect size (d) of 0.47 for depression, a significance level of 0.05 (one-tailed), and a statistical power of 0.80, a sample of at least 30 people was required. This study therefore recruited 48 couples, of which 11 couples either withdrew from the study or did not present to a scheduled appointment) and four couples gave birth during the intervention period, meaning that 33 couples participated in the pretest, the intervention program, and the posttest (Figure 3). This study followed the Transparent Reporting of Evaluations with Nonrandomized Designs (TREND) statement [21] to improve the reporting quality of nonrandomized evaluations of behavioral interventions.

### Intervention program

#### Program development

A couple-centered antenatal education program was developed to help couples’ adaptation to become parents and their relationship. The environmental interaction model [22] of the process of becoming a mother was used as a theoretical basis for the educational program. A couple-centered psychoeducation method was selected according to the results of a previous study [23]. Through a critical evaluation of various studies [16-18,20,24-29] of effective nursing interventions to help parental adaptation and couple relationships, the content and methods of the program were specifically constructed. In order to confirm the validity of the content of the educational program, the initially developed program was reviewed by experts, including nurses and midwives in women’s hospitals, maternity and women’s health nursing professors, and women’s health researchers. In addition, after listening to the opinions of women who had experienced pregnancy and childbirth within the past 3 years, content related to the husband’s pregnancy experience and massage was added to the intervention program (Figure 2).

#### Program content

The couple-centered antenatal education program applied in this study (Table 1) had two main content areas: adaptation of the couple relationship [16-17,23] and becoming parents for couples [13,22]. The content on couple-relationship adaptation included emotional intimacy, support, respect, empathy, gratitude, and emotional exchange between couples from pregnancy to the postpartum period as ways to deal with marital problems and difficulties. The content dealing with becoming parents included promoting well-being in response to physical and mental changes, discomfort, depression, and stress, self-care, parental-fetal attachment, preparation for normal delivery, and preparation for the parenting role (e.g., newborn baby care and breastfeeding). Each session set 2 to 3 specific goals for couples, such as social-role preparation, instructions on infant caregiving, and the promotion of fetal attachment, self-care, and well-being.

#### Application of the program

Two nurses (a women’s health nurse and a midwife/breastfeeding expert) provided the educational program in four sessions, each of which lasted for 1 hour in the childbirth education room of the hospital. The size of each intervention group was decided based on couples’ needs, with the education provided to one to three couples at a time. The teaching method consisted of face-to-face education and counseling. Throughout the session, we provided education and information to help the couples discuss these topics, allocated time to practice skills such as expressing one’s own emotions and effective conversation techniques, and provided time for questions and answers. In addition, a small gift was presented to encourage participants to continue participating in the program, and periodic text messages were sent as reminders before scheduled appointments and to express appreciation for the participants’ participation.

### Measurements

The study variables were measured before and after the educational program to test the program’s effectiveness. The researcher explained to the couple how to answer each questionnaire and asked them to respond separately to prevent couple-related dynamics from affecting the responses. The measures for adaptation to becoming parents were antenatal depression and stress for the parents themselves, parental-fetal attachment for the parental-fetal relationship, newborn-care confidence for becoming parents, and fear of childbirth for mother’s childbirth preparation, while dyadic adjustment was used to measure adaptation of the couple relationship.

#### Prenatal depression

Depression in pregnant couples was measured using the Korean version of the Edinburgh Postnatal Depression Scale [30], which was translated by Kim et al. [31] and is one of the most widely used tools for assessing perinatal depression in both women and men [32]. The scale contains 10 items. Each item is scored on a 4-point Likert scale (0, no; 3, most of the time), and the total score thus ranges from 0 to 30. Scores above 10 are considered to indicate depression in Korea [31]. The reliability of the original instrument was indicated by a Cronbach’s α of 0.87 [30]; in the present study, Cronbach’s α was 0.77–0.78 in the pregnant women and 0.56–0.63 in their husbands.

#### Stress

Stress in pregnant couples was measured using the Korean version of the Perceived Stress Scale [33], which was translated by Park and Seo [34]. The scale contains 10 items. Each item is scored on a 5-point Likert scale (0, never; 4, very often); thus, the total score ranges from 0 to 40. The reliability of the original instrument was indicated by a Cronbach’s α of 0.84–0.86 [33]; in the present study, Cronbach’s α was 0.78–0.83 in the pregnant women and 0.79–0.82 in their husbands.

#### Parental-fetal attachment

Parental-fetal attachment was measured using the Korean version of the Maternal–Fetal Attachment Scale [35], which was translated by Kim [36]. The scale contains 25 items. Each item is scored on a 4-point Likert scale (1, never; 4, always) and the total score therefore ranges from 25 to 100. A higher score indicates a higher degree of fetal attachment [36]. Since this is a tool for measuring maternal-fetal attachment, paternal attachment was measured by reframing questions 22, 23, and 24 for a husband as dealing with pride and concern about his pregnant wife [36]. The reliability of the original tool was indicated by a Cronbach’s α of .84 [35]; in the present study, Cronbach’s α was 0.94-0.95 for maternal-fetal attachment and 0.93–0.94 for paternal-fetal attachment in the present study.

#### Newborn-care confidence

Confidence in caring for newborns was measured using a self-report questionnaire [37]. The 22-item scale is scored on a 5-point Likert scale (0, very uncertain; 5, very confident), and the total score thus ranges from 22 to 110. A higher score indicates higher confidence in raising newborns. The reliability of the original instrument was indicated by a Cronbach’s α of 0.84 [37]; in the present, Cronbach’s α was 0.94–0.96 in the pregnant women and 0.95–0.97 in their husbands.

#### Maternal fear of childbirth

Childbirth fear in pregnant women was measured using the Korean version of the Wijma Delivery Expectancy Questionnaire (version A) [38] before delivery, which was translated by Park et al. [39]. The scale contains 33 items, each of which is scored on a 5-point Likert scale (1, not at all; 5, extremely); therefore, the total score ranges from 33 to 165. Higher scores indicate greater fear. The reliability of the original instrument was indicated by a Cronbach’s α of 0.87 [38]; in the present study, Cronbach’s α was 0.91–0.93.

#### Couple’s dyadic adjustment

Marital adaptability was measured using the Dyadic Adjustment Scale (DAS)-10 [40], which is a shortened Korean form of the DAS reported by Cho, Choi, Oh, and Kwon [41] that has demonstrated reliability and validity. The scale contains 10 items. Items are scored on a 5- or 6-point Likert (0: not at all; 5: always; and/or 6: perfectly), and the total score thus ranges from 0 to 51. Higher scores indicate higher marital satisfaction, and a cut-off point is 32 or higher is used as a categorical measure of marital satisfaction [40]. The reliability of the DAS-10 was indicated by a Cronbach’s α of 0.83 [41]; in the present study, Cronbach’s α was 0.77–0.83 in the pregnant women and 0.68–0.83 in their husbands.

#### General characteristics

Information was gathered on participants’ age, educational background, occupation, marriage duration, family income, whether the pregnancy was planned, gestational age, and lactation plan.

#### Program satisfaction survey

Participants were asked about their satisfaction with the overall program content, the topic that they most enjoyed among the four sessions of the program, any material that they thought was missing or could have been supplemented, perceptions regarding any other educational content that would be needed in pregnancy and childbirth education, and the educational content that they desired in postnatal education programs.

### Statistical analysis

The effect of the intervention in a single group was analyzed using the one-tailed paired t-test using IBM SPSS ver. 25.0 (IBM Corp., Armonk, NY, USA).

## Results

### General characteristics

The pregnant women were 32.30±3.10 years of age (mean±standard deviation), and their husbands were 33.21±6.25 years of age. Almost all of the pregnant women (97.0%) and all of their husbands had at least a college education, 72.7% of the pregnant women and all of their husbands were employed, and 42.4% of the families had a monthly family income of 4–5 million Korean won (approximately 3,500-4,400 US dollars). The average marriage duration was 2.34±1.63 years, the average gestational age at the time of recruitment was 31.30±2.66 weeks, and 78.8% of the participants had a planned pregnancy (Table 2).

### Changes in parental adaptation and dyadic adjustment of couples

#### Prenatal depression and stress

The score for antenatal depression of the pregnant women decreased significantly from 6.12±3.67 before the intervention to 5.18±3.62 after the intervention (t=-1.89, *p*=.034). The score of the husbands decreased from 3.73±2.52 to 3.67±2.88, but this change was not significant (t=-0.10, *p*=.460). The stress score of the pregnant women decreased significantly from 15.30±5.48 to 13.33±5.75 (t=-2.18, *p*=.018), while their husbands showed a non-significant decrease from 12.70±4.66 to 12.27±5.05 (t=-0.53, *p*=.300).

#### Parental-fetal attachment

The score for maternal-fetal attachment increased significantly from 73.30±13.37 to 77.67±12.86 (t=3.28, *p*=.001), as did that for paternal-fetal attachment, from 79.85±12.18 to 84.30±10.02 (t=3.06, *p*=.002).

#### Newborn-care confidence

The score for confidence in providing newborn care increased significantly from 73.27±12.42 to 78.58±12.51 in the pregnant women (t=4.02, *p*<.001) and from 79.88±17.83 to 83.39±12.78 in their husbands (t=1.85, *p*=.037).

#### Maternal fear of childbirth

Pregnant women’s score for fear of childbirth decreased significantly from 68.97±19.76 before the intervention to 63.45±20.01 after the intervention (t=-2.12, *p*=.021).

#### Couples’ dyadic adjustment

The score for marital adaptability increased from 41.06±5.28 before the intervention to 41.86±4.12 after the intervention among pregnant women, but this change was not statistically significant (t=1.30 *p*=.101). Their husbands’ scores decreased by 0.67±3.81, from 43.88±3.28 before the intervention to 43.21±4.64 after the intervention, but this change was likewise not statistically significant (t=-1.01, *p*=.161) (Table 3).

### Program adherence and applicability

Over the four sessions, two of the 33 couples were absent once, due to the difficulty of moving to another city and the poor condition of the pregnant woman on the day of the session. Educational videos were provided to the couples who were absent, and after watching they gave their impressions as well as their answers to questions. The program completion rate was 100% when evaluated based on the pregnant couples participating in three of the four sessions, corresponding to a 78% attendance criterion.

After completing the antenatal education program, 50.0% of the participants were very satisfied, and 41.4% were satisfied. Satisfaction with the educational topics was highest for newborn care (54.8%), followed by preparation for childbirth (25.0%), communication within couples (12.4%), and adaptation to the pregnancy and postpartum periods (7.8%).

## Discussion

### Development of the couple-centered antenatal education program

This study is the first in Korea to apply couple-centered education in mainstream antenatal education to help adaptation to parenthood in terms of both the marital relationship and the parental role. A unique aspect of the program is that the educational content was well-balanced in terms of adaptation to the parental role and interactions between spouses. In addition, the present study contributes to the expansion of nursing theory and the formation of a body of knowledge in nursing science by constructing a theoretical model of becoming parents for pregnant couples based on the theory of becoming a mother and the intervention effects [22].

The content related to parental adaptation dealt with physical, emotional, and relational changes during pregnancy and postpartum, childbirth preparation, and newborn care. Content related to maintaining the couple relationship was also included, since the couple relationship serves as an important psychological basis for the couple to perform the parental role together [42]. This enabled the pregnant women and their husbands to prepare for becoming parents during pregnancy, while meeting the demands of pregnancy, childbirth, and the postpartum period. This approach reflects the fact that fathers are also active in preparing for parental change and have increased expectations [10,12]. Accordingly, the beneficiaries of education were both mothers and fathers, and the program was designed to help them maintain a mutually supportive relationship through dialogue and sympathy for understanding each other and physically and mentally preparing for parenthood.

The education on couple-relationship adaptation included emotional intimacy, support, respect, empathy, and emotional exchange between partners after pregnancy and childbirth. This program was offered at an appropriate time for couples to participate together. On-site participation was chosen based on evidence that this setting is effective and that participants prefer to interact with educators, have vivid experiences, and get answers to questions immediately [21,43]. The antenatal education program was administered to small groups of at most three couples per session, which satisfied their individual needs, facilitated active counseling and conversation techniques, and allowed sufficient interactions with the participants [43,44].

### Effects of the couple-centered antenatal education program

The effects of this couple-centered antenatal education program were analyzed in terms of variables for confirming parental adaptation: antenatal depression, antenatal transition stress, parental-fetal attachment, newborn-care confidence, and fear of childbirth. Pregnant women who participated in the program showed reductions in antenatal depression, stress, and fear of childbirth, as well as improvements in maternal-fetal attachment and confidence in providing newborn care. Their husbands showed improvements in attachment with the fetus and confidence in providing newborn care. This is consistent with previous reports that pregnant couples who participated in antenatal education showed increased confidence in parental adaptation (e.g., childbirth and parenting), and increased communication and cooperation with their husbands to overcome anxiety and stress [45,46]. In the present study, only the pregnant women (i.e., not their husbands) showed reduced depression and stress. This contrasts with a report of improved mental health among couples who participated in a marital-relationship program during pregnancy, a time of transitioning to parenthood [47]. This discrepancy is attributable to the levels of depression and stress being very low in the husbands, indicating a floor effect. This is similar to the report of Petch and Halford [29], who explained that among couples who received parental-role education and marital-relationship education, there was no effect of education when parental-role stress was low. The finding of a significant reduction in maternal fear of childbirth is consistent with previous findings [48]. In light of the result that marital adaptation is a predictor of fear of childbirth [2], this program included not only childbirth-preparation education in order to reduce fear of childbirth but also education to increase marital adaptation. It is believed that confidence in childbirth will increase and the fear of childbirth will decrease in couples who receive education that prepares them for childbirth and helps them to maintain a good couple relationship.

However, there was no effect on marital adaptation. The score of marital adaptation was 43 and 41 for the pregnant women and their husbands, respectively, in this study, compared with a threshold score of 32 reported by Cho et al. [41]. A recent meta-analysis reported a small improvement in marital-relationship satisfaction [17], but the marital adaptation score after the educational intervention remained similar to that before the intervention, perhaps because the marital-relationship education helped couples to feel satisfied with their current marital relationship in terms of having positive feelings and preventing future marital problems [49]. In the pre- and postexamination of this study, both the pregnant women and their husbands had a marital adaptation score of more than 80% of the total score of 50 points, and there was no significant difference between the pretest and postscores, so it seems that satisfaction in marital adaptation was maintained. The lack of an observed improvement in the marital relationship underscores the need to reexamine the effectiveness of this aspect of the program through a follow-up investigation.

This study only confirmed the antenatal effects of couple-centered education aiming to help participants become parents in a single group, but a recent meta-analysis showed that couple psychoeducation reduces maternal postpartum depression and ameliorates paternal negative affect, as well as leading to overall improvements in couples’ relationship satisfaction [17]. Therefore, future studies will need to confirm the effect of the intervention by expanding it to the postpartum period with a control group. In addition, a single-group pre-post experimental study was conducted to evaluate the applicability of the program; therefore, the degree to which the effectiveness of the program is limited because it was not possible to control for confounding variables and maturity issues. In subsequent studies, single-blind randomization is required for more reliable results by establishing both a control group and an experimental group. Another limitation of this study is that the confidence intervals were relatively wide due to the small sample size. As a general rule, as a sample size increases, the confidence interval should become narrower. Therefore, the effectiveness of this intervention should be examined with a larger sample. Further, the participants in this study consisted of highly educated couples with a good marital relationship in their first pregnancies, limiting the generalizability of the results to couples with other demographic characteristics.

Future studies should consider combining online and offline education in order to reduce the discontinuation of participation related to temporal and spatial restrictions. In the present antenatal education program, scores of higher than 90% were found for both parental adaptation in caring for newborns and preparation for childbirth, and also the satisfaction of the couples with their adaptation during This couple-centered antenatal education program based on the model of becoming parents seems to be effective and feasible as a way to promote the adjustment of pregnant couples to parenthood. Antenatal education programs should be implemented with both couple-relationship and parental adaptation elements to help pregnant couples adjust to parenthood. Future studies should address the need for programs to reflect educational demands related to fathers’ preparations for becoming parents and caring for newborns.

## Figures and Tables

**Table 1.** The couple-centered antenatal education program

Goal based on the ecological interaction model	Methods	Content
**Session 1: Becoming happy parents (1 hour by a midwife)**		
Social-role preparation	Education, information, practice, Q&A	Happy environment, relieving stress, and participation of the husband
Attachment promotion: parental-fetal attachment		Talking and singing with finger movement for the fetus
Self-care promotion: preparation for a normal delivery	Education, information, practice, Q&A	Normal delivery process and husband roles
		Method of how to breathe, relax, and massage to relieve the pain
**Session 2: Becoming competent parents (1 hour by a midwife)**		
Social-role preparation	Education, information, Q&A	Newborn care in growth and development, normal reflex reactions, and getting used to baby signals, father–child care
Infant-caregiving instruction	Education, information, Q&A	Advantages of breastfeeding (for mother and baby), and breastfeeding method, baby’s signals, and deep breastfeeding (latch and positioning)
	Practice, Q&A	Breast massage and breastfeeding, holding a baby, feeding and burping, soothing a crying baby, changing diapers, bathing, and managing the umbilical cord
**Session 3: Becoming a happy couple and family (1 hour by a women’s health nurse)**		
Well-being promotion	Education, information, conversation, Q&A	1. Physical and mental changes in the pregnant woman and her husband
		2. Check the mother’s and father’s situations during the perinatal period and talk about each other’s difficulties and how to help, and how to maintain the marital relationship
	Experience	1. Husbands wear pregnancy simulator vest
	Conversation practice	1. Identify usual communication patterns, and practice positive self-assertive communication
Self-care promotion	Education, information, Conversation, Q&A	1. Physical changes, and discomfort of pregnant women, and caution and self-care methods, importance of spousal support during the third trimester
		2. Prenatal and postpartum depression, stress related to the parental transition in couples, the importance of mutual support for couples, and self-care for couples, express their feelings with each other on parental transition
**Session 4: Becoming a happier couple (1 hour by a women’s health nurse)**		
Well-being promotion	Education, information, conversation practice, Q&A	1. Understanding and empathy between spouses, and the effects of the virtuous cycle of expressing gratitude and empathy
		2. Identifying common marital problems in parents and discuss current marital problems or difficulties
		3. Characteristics of well-adapting parents, the positive effects of hugging, and consideration for each other
	Conversation practice	1.Thank-you messages (conversations and letter-writing)
		2.Talk about what they want from others
Social-role preparation	Education, information, conversation Q&A	1. Importance of accepting and adapting to parenthood in a positive manner
		2. Talking about the difficulties, burdens, and fears of childbirth

**Table 2.** Demographic characteristics of the pregnant couples (N=33)

Variable	Categories	n (%)	Mean	SD
Pregnant woman				
Age (years)			32.30	3.10
Education	High school	1 (3.0)		
	University	26 (78.7)		
	Graduate school	6 (18.3)		
Employed	Yes	24 (72.7)		
	No	9 (27.3)		
Monthly family income (KRW)	<2 million	2 (6.1)		
	2–3.99 million	12 (36.4)		
	4–5.99 million	14 (42.4)		
	≥6 million	5 (15.2)		
Marriage (years)			2.34	1.63
Gestation (weeks)			31.30	2.66
Planned pregnancy	Yes	26 (78.8)		
	No	7 (21.2)		
Husband				
Age (years)			33.21	6.25
Education	University	26 (78.8)		
	Graduate school	7 (21.2)		
Employed	Yes	33 (100)		
	No	0 (0)		

KRW: Korean won (1 million KRW is approximately 900 US dollars).

**Table 3.** Effectiveness of the couple-centered antenatal education intervention (N=33)

Variable	Pretest		Posttest		Difference		95% confidence interval	t	*p* (one-tailed)
	Mean	SD	Mean	SD	Mean	SD			
Pregnant women									
Prenatal depression	6.12	3.67	5.18	3.62	-0.94	2.86	-0.10 to -1.78	-1.89	.034
Prenatal stress	15.30	5.48	13.33	5.75	-1.97	5.19	-0.13 to -3.81	-2.18	.018
Newborn-care confidence	73.27	12.42	78.58	12.51	5.30	7.58	2.61 to 7.99	4.02	<.001
Maternal-fetal attachment	73.30	13.37	77.67	12.86	4.36	7.64	1.65 to 7.07	3.28	.001
Fear of childbirth	68.97	19.76	63.45	20.01	-5.52	14.94	-0.22 to -10.81	-2.12	.021
Couple’s dyadic adjustment	41.06	5.28	41.85	4.12	0.79	3.47	-0.44 to 2.02	1.30	.101
Husbands									
Prenatal depression	3.73	2.52	3.67	2.88	-0.06	3.44	-1.28 to 1.16	-0.10	.460
Prenatal stress	12.70	4.66	12.27	5.05	-0.42	4.60	-2.05 to 1.21	-0.53	.300
Newborn-care confidence	79.88	17.83	83.39	12.78	3.52	10.93	0.32 to 6.72	1.85	.037
Paternal-fetal attachment	79.85	12.18	84.30	10.02	4.45	8.35	1.49 to 7.42	3.06	.002
Couple’s dyadic adjustment	43.88	3.28	43.21	4.64	-0.67	3.81	-2.02 to 0.68	-1.01	.161
